# Polarization of Magnetoplasmons in Grating Metamaterials Based on CdTe/CdMgTe Quantum Wells

**DOI:** 10.3390/ma13081811

**Published:** 2020-04-11

**Authors:** Dmitriy Yavorskiy, Maria Szoła, Krzysztof Karpierz, Rafał Rudniewski, Rafał Bożek, Grzegorz Karczewski, Tomasz Wojtowicz, Jerzy Wróbel, Jerzy Łusakowski

**Affiliations:** 1 Faculty of Physics, University of Warsaw, ul. Pasteura 5, 02-093 Warsaw, Poland; 2 Centera Laboratories, Institute of High Pressure Physics, Polish Academy of Sciences, ul. Sokołowska 29/37, 01-142 Warsaw, Poland; 3 Institute of Physics, Polish Academy of Sciences, Aleja Lotników 32/46, 02-668 Warsaw, Poland; 4 International Research Centre MagTop, Institute of Physics, Polish Academy of Sciences, Aleja Lotników 32/46, 02-668 Warsaw, Poland

**Keywords:** THz spectroscopy, metamaterials, polarization, magnetoplasmons

## Abstract

Grating metamaterials were fabricated with electron beam lithography on CdTe/CdMgTe modulation doped structures with two non-interacting quantum wells. Two types of samples were studied: with etched gratings and with gratings formed by deposition of Au stripes. The polarization properties at THz frequencies of the gratings were determined at room temperature. It was shown that Au gratings formed a linear polarizer, while etched gratings did not polarize THz radiation. Transmission of circularly polarized THz radiation at low temperatures through a sample with no grating showed a strongly circularly polarized cyclotron resonance transition. Transmission of this radiation through a sample with an etched grating showed a magnetoplasmon transition that was almost perfectly linearly polarized. We concluded that magnetoplasmons in metamaterials with etched gratings are linearly polarized excitations, possibly with a small contribution of a circular component. This work opens the possibility of the detailed study of the polarization of magnetoplasmons, which has not been explored in the past.

## 1. Introduction

Metamaterials comprise a broader and broader spectrum of artificial materials fabricated with different physical and chemical methods [1,2]. In the case of semiconductors and dielectrics, one finds lithographically-defined structures as the most abundant examples of such artificial materials. In particular, periodic lattices of split-ring resonators are often used as metamaterials with a relatively easy means of fabrication, flexibility of design, and tunability [3,4,5]. A feature of such metamaterials is that their resonances typically fall within a broad frequency band from GHz to THz [6,7].

Cadmium telluride-based metamaterials are an interesting option in modern technologies. It seems that the main reason for this interest is related to the application of CdTe-based compounds in solar cells, where the efficiency of light to electric power conversion has been continuously growing [8] and for which quantum efficiency has already surpassed 80% [9]. What is more, metamaterials based on CdTe allow increasing the absorption of light, which is crucial for efficient harvesting of energy [10,11].

The present paper is concerned with a special type of metamaterial, which has the form of a semiconductor structure on which a grating was lithographically fabricated either by etching of the surface or by evaporation of a metal. Metamaterials of this type were recently studied in a number of papers [12,13,14,15,16,17,18]. A distinguishing feature of the samples studied is the presence of a two-dimensional electron gas (2DEG) of a very high mobility of electrons at low temperatures. This feature allowed us to analyze the excitation of the cyclotron resonance (CR) and magnetoplasmons (MPs) at THz frequencies, low temperature (*T*), and high magnetic field (*B*).

The following remarks will allow us to put the results of this research in a more general context. If one considers the momentum of a THz photon with the frequency $\omega_{l}$ = 2π×2.52 THz (a radiation of this frequency was mostly used in the present study), then the photon momentum is $\hbar \omega_{l}/c\approx 5,\;6\times 10^{-30}$ Ns. In semiconductors, the wavelength $\lambda_{p}$ of a plasmon with a frequency $\omega_{p}$ of about 1 THz is typically of the order of a few μm, so an estimate of its momentum gives $2\pi \hbar /\lambda_{p}∼10^{-28}$ Ns, which is about two orders of magnitude larger than the momentum of a THz photon. Due to the large mismatch of momenta, a periodic structure (typically, a grating) is prepared on the surface of a studied sample. Then, in the near-field (i.e., close to the grating), the electromagnetic field of the incident wave carries spatial Fourier components with wave vectors given by $k_{n}=2n\pi /\Lambda$, where Λ is the period of the grating and *n* is an integer. Thus, with a grating with the period of the order of 1 μm, one can fulfill the principle of the conservation of momentum in the process of the creation of a plasmon with a THz photon. Such gratings have been routinely used in studies of plasma in solids, beginning with the first study of plasmons in a 2DEG by Allen et al. in 1977 [19].

To get a spectrum of plasmonic excitations at B=0, one needs to tune the frequency of the incident radiation and monitor the response of the system (transmission, photovoltage, etc.). This is typically done in Fourier spectroscopy [19] or in time-domain spectroscopy [12,13] experiments. In the case of a 2DEG, one can use also a semi-transparent gate to tune the concentration of 2DEG ($n_{s})$ and thus to change its plasmon frequency since $\omega_{p}∼\sqrt{n_{s}}$.

The magnetic field introduces essential changes to the propagation of electromagnetic waves in conductive media. It is far beyond the scope of this paper to give a detailed account of this subject. The interested reader is encouraged to address classical books [20,21] describing these issues in the three- and two-dimensional cases, respectively.

To continue, we have to define the system to be studied more precisely. We will consider a 2DEG residing in a modulation doped quantum structure, with the plane of the 2DEG perpendicular to the external magnetic field. A grating is prepared on the surface of the sample, and the laser radiation (frequency $\omega_{l}$) is incident normal to the 2DEG plane, thus parallel to *B*. Due to the incident radiation, (magneto)plasmons are excited in the 2DEG, which propagate in the plane of the 2DEG, at a right angle to *B*, i.e., corresponding to the Voigt configuration. In this case, one finds that the frequency of an MP resonance $\omega_{mp}$ fulfills the relation [20,21]:

(1) $$\omega_{mp}^{2}=\omega_{p}^{2}+\omega_{c}^{2},$$

where the cyclotron resonance frequency $\omega_{c}=eB/m$, with *e* and *m* being the electron charge and effective mass, respectively. An MP resonance is excited with the laser radiation, so for the given frequency of laser photons, it appears at *B* for which $\omega_{mp}=\omega_{l}$.

The problem of the polarization of magnetoplasmons is not trivial since the cyclotron resonance is circularly polarized (i.e., it is active in only one circular polarization), and the plasmon itself is a longitudinal excitation. It cannot be solved just by placing a circular polarizer in the path of the incident beam because a metallic grating acts as a linear polarizer. For example, measurements presented in [13] showed an extinction ratio of a grating-metamaterial on a GaN/AlGaN heterostructure to be more than 1:80.

A solution to this problem in the case of MPs in a 2DEG is shown in the present paper and is given by the application of etched gratings. As is shown below, etched gratings allow for the excitation of MPs (they allow for the generation of adequate spatial Fourier components of the electromagnetic field), but at the same time, they do not act as linear polarizers.

The paper is organized as follows. Section 2 describes the samples used, the experimental techniques, and the setups. The results are presented in Section 3 and are discussed in Section 4.

## 2. Samples and Experiment

**Figure 1:**
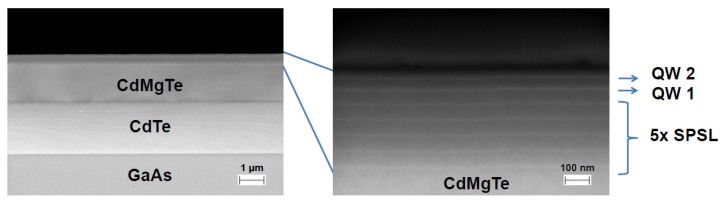
SEM photographs of the layered structure of the sample studied. QW1 and QW2 are the quantum wells. SPSL, short period superlattice.

Cadmium telluride-based quantum structures used in the present study were grown by molecular beam epitaxy on a semi-insulating GaAs substrate on which a buffer layer was grown. The buffer layer consisted of a 2.2 μm thick CdTe, a 1.5 μm thick CdMgTe layer, and 5 repetitions of a short period superlattice (SPSL; each period consisted of 10 repetitions of 2 monolayers of CdTe and 2 monolayers of CdMgTe, separated by a 52 nm thick CdMgTe spacer). Next, two 10.5 nm thick modulation doped CdTe quantum wells (QWs) separated by 44 nm thick CdMgTe barrier were grown. The growth was finished with a 45 nm thick CdMgTe cap layer. The magnesium content in all CdMgTe layers was equal to 26%. Modulation doping with iodine donors resulted in formation of a 2DEG in the wells. The concentration of electrons was estimated by magnetotransport measurements to be equal to about $4.7\times 10^{11}$ cm^−2^ in each well. Figure 1 shows the details of the layered structure of the sample obtained with a scanning electron microscope (SEM).

The gratings were patterned with electron beam lithography. We applied a low-temperature procedure, which has been shown to be preferable in the case of II-VI materials [22]. For this reason, instead of using a standard PMMA resist, we applied a positive-tone resist CSAR 62, for which the baking temperature is lower than that for PMMA. We found that baking the samples on a hot plate for 5 min at 155 °C gave satisfactory results. In the case of wet etching applied in this study, a strong adhesion of the resist mask (with a thickness of about 100 nm) to the substrate was required, and this was achieved with the procedure described. A solution of bromium in glycol ethylene (volume proportion 3:5) was used, and etching was stopped in methanol. Gold gratings were patterned with CSAR 62 resist masks. A Au/Cr layer (with the thickness of Au and Cr equal to about 40 nm and 10 nm, respectively) was thermally evaporated, and gratings were finally obtained with a lift-off technique. The thickness of the Au and Cr layers was estimated on the basis of the known calibration of the vacuum sputter used.

**Figure 2:**
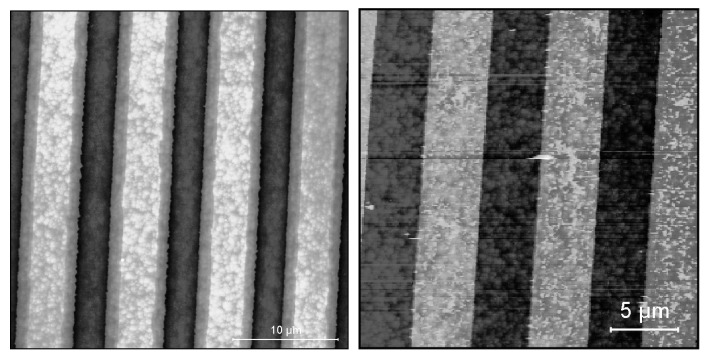
AFM photographs of the surface of samples with etched (**left**) and evaporated (**right**) grating. The period of grating is equal to 8 μm in both cases.

Examples of atomic force microscope (AFM) photographs of the surface of samples after etching or evaporation of the grating are shown in Figure 2. In the case of Au/Cr gratings, we tested samples with the grating period Λ equal to 2 μm, 4 μm, 8 μm, and 16 μm. In the case of etched gratings, we studied samples with the grating period equal to 8 μm and the depth of etching *D* changing from 20 nm to 300 nm. In each case, the geometrical aspect ratio of the grating was close to 50%. In the case of a metallic grating, the geometrical aspect ratio was the fraction of Λ covered with the Au bar. In the case of etched gratings, it was the fraction of Λ that was not etched.

**Figure 3:**
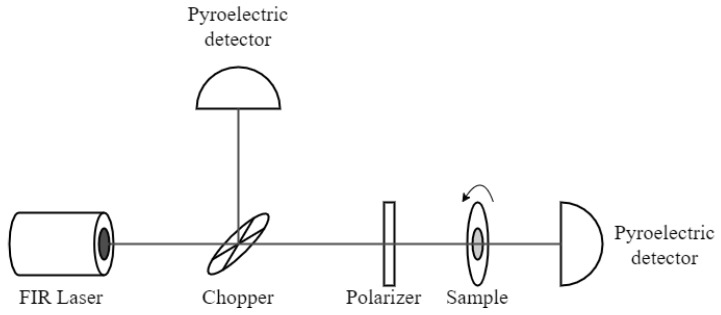
An experimental setup for testing the polarization properties of samples at room temperature.

The polarization properties of gratings were tested at room temperature in an experimental system shown in Figure 3. The source of radiation was an optically pumped molecular laser (FIRL-100 from Edinburgh Instruments Ltd., Livingston, UK). A beam from the laser was directed to a rotating wheel of a mechanical chopper. A beam reflected from the wheel was incident on a pyroelectric detector (Thorlabs Inc., Newton, NJ, USA), which allowed us to monitor the power of the laser; this signal was subsequently used to normalize the data. If necessary, a circular polarizer was put in front of the samples. The sample itself was placed on a rotating support, and the intensity of transmitted radiation was registered with another pyroelectric detector. Measurements were carried out as a function of the angle of rotation of the support and normalized to the power of the laser. The angle in polar plots presented below defines the orientation of grating bars with respect to the horizontal direction, i.e., when the bars are horizontal, the angle is equal to 0. Polarization of the laser radiation was vertical; that was why the maximum transmission for a metallic grating was with the bars oriented at 90°.

Excitation of the CR and MPs was done with the sample cooled to 2 K and placed in the center of a superconducting coil (the cryostat with a variable temperature insert and the coil were from Cryogenic Ltd., London, UK). A carbon bolometer placed behind the sample allowed us to measure the intensity of the transmitted radiation as a function of the magnetic field. The bolometer was just a mechanically thinned Alan–Bradley resistor. A circular polarizer (composed of a λ/4 quarter plate made of a crystalline quartz and a linear polarizer) was placed in front of the sample, and we measured the transmitted signal in $\sigma^{+}$ and $\sigma^{-}$ polarizations by inverting the direction of the magnetic field.

The transmission of the radiation of a wavelength equal to 96 μm, 118.8 μm, 164 μm, and 186 μm through the sample at low temperature and as a function of the magnetic field was registered. In particular, the observation of the CR transition in the unprocessed sample allowed us to determine the effective mass of the electron equal to 0.103$m_{0}$ ($m_{0}$ is the mass of free electron). For polarization measurements, we concentrated on 118.8 μm radiation only due to the high power and long-term stability of the power of the laser at this wavelength.

In each case, signals were registered with a lock-in technique, with the phase-sensitive amplifiers referenced by the chopper.

## 3. Results

**Figure 4:**
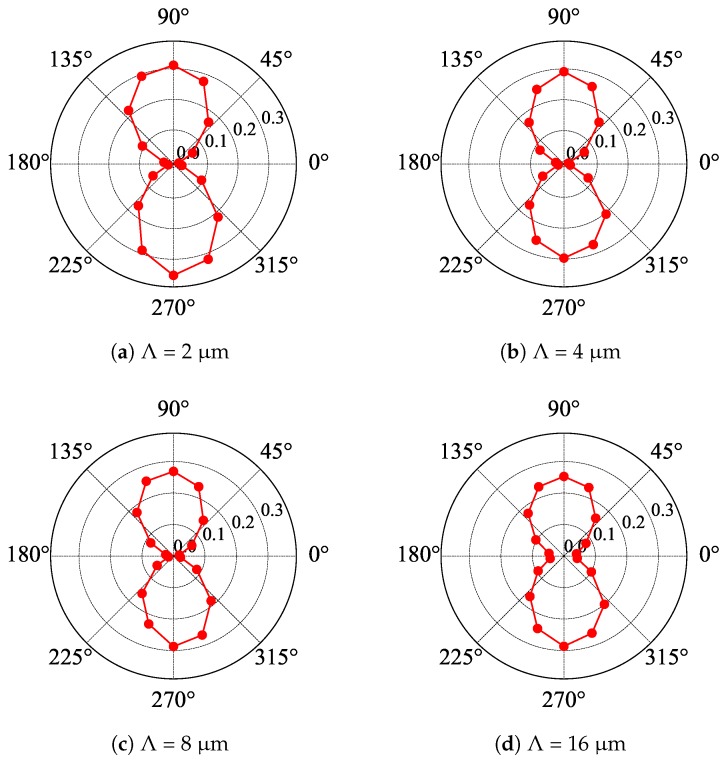
Polar plots of the transmission of linearly polarized radiation with the wavelength equal to 118.8 μm through samples with Au gratings of period Λ. An angle of zero corresponds to the horizontal orientation of the grating’s bars. The laser radiation is linearly polarized in the vertical direction.

**Figure 5:**
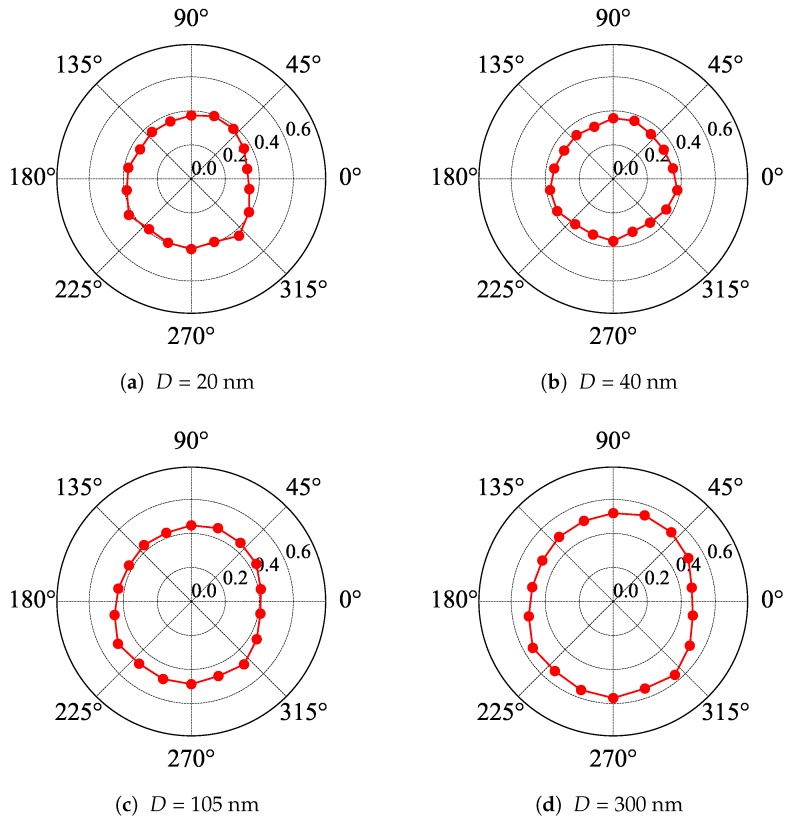
Polar plots of the transmission of linearly polarized radiation (the wavelength equal to 118.8 μm) through samples with etched gratings of a period equal to 8 μm and the depth of grooves *D*. An angle of zero corresponds to the horizontal orientation of the grating’s bars. The laser radiation is linearly polarized in the vertical direction.

The results of the polarization measurements carried out at room temperature are shown in Figure 4 and Figure 5 for samples with Au and etched gratings, respectively. It was evident that Au gratings acted as linear polarizers, while etched gratings did not. The extinction ratio for Au gratings was estimated to be equal to 1:55, 1:46, 1:18, and 1:7 in the case of Λ equal to 2 μm, 4 μm, 8 μm, and 16 μm, respectively. One could conclude that for the radiation with the wavelength equal to 118.8 μm, the two first gratings formed quite a good linear polarizer, while this was not the case for larger Λ. Notice that in other polarization studies, to have the radiation linearly polarized, we typically would use a Au grating evaporated on a mylar foil with a density of 500 or 1000 lines per inch, which corresponds to the period of approximately 2–5 μm and an extinction ratio of about 1:100. Thus, it is not surprising that short-period Au gratings act as good linear polarizers. The results for small-Λ gratings were only slightly worse than those reported in [13].

Note also that numerical simulations and experiments showed (see, e.g., [23,24]) that the extinction ratio of wire grating polarizers depended on the details of their construction: the material used, the presence (or not) of a substrate, the period of the grating, and the thickness of the wires. Generally, for a given wavelength of radiation, the extinction became worse and worse when the period of the grating increased. This was exactly the effect that we observed in our experiment.

**Figure 6:**
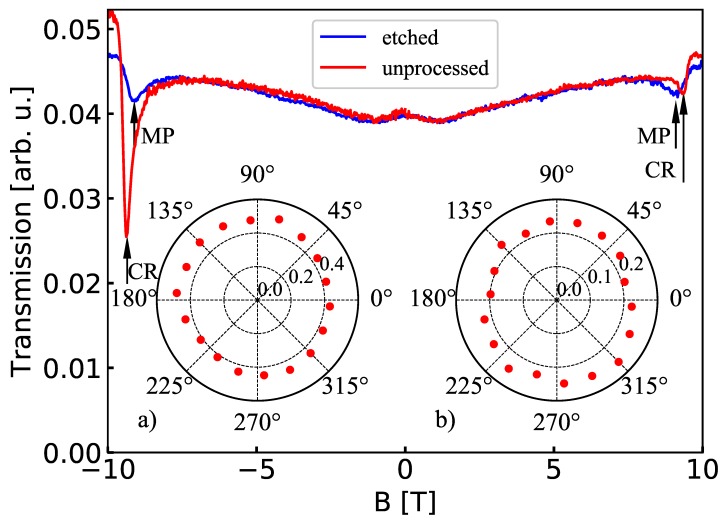
Transmission spectra (at 2 K) through an unprocessed sample (red) and through the sample with the etched grating with *D* = 105 nm and Λ = 8 μm. CR and MP resonances are marked with arrows. Insets (**a**,**b**) show the transmission (at 300 K) of linearly and circularly polarized radiation through the sample with the etched grating with *D* = 105 nm. An angle of zero corresponds to the horizontal orientation of the grating’s bars. The laser radiation is linearly polarized in the vertical direction.

To confirm the lack of the polarizing properties of the etched gratings, we present in Figure 6a comparison of the transmission of linearly and circularly polarized radiation through the sample with *D* = 105 nm (Insets (a) and (b), respectively). As it should be in the case of a non-polarizing element, the intensity of the transmitted signal did not depend on the angle of rotation of the sample.

The results of the polarization measurements at low temperature are presented in Figure 6. In the case of an unprocessed sample (without any grating), we observed a resonance due to the excitation of the CR. In theory, this transition should be fully circularly polarized, which was not observed in the case of the results presented in Figure 6. We attributed this difference to imperfections in the optical path, which could result from the imperfections of the circular polarizer, a non-planar front of the wave propagating between the source and the detector, or a slight tilt of the sample. Nevertheless, a strong circular polarization of the CR in the case of the unprocessed sample was evident.

The situation was quite different in the case of the sample with the etched grating, which showed a transition that was not circularly polarized (blue curve in Figure 6). A shift in the position of the resonance, with respect to the position of CR, indicated that we are dealing with the excitation of a magnetoplasmon. Based on this result, we proposed that the magnetoplasmons in the studied sample with the etched grating were linearly polarized.

## 4. Discussion

The path of argumentation leading to the above conclusion was the following. First, we showed that etched gratings (as opposed to Au ones) did not show any polarization properties, which meant that circularly polarized radiation, incident on such a grating, did not change its polarization during transmission. Second, we showed that we were able to generate and transmit circularly polarized radiation to the sample cooled to a low temperature, which is confirmed by the red curve in Figure 6. Third, based on earlier studies on magnetoplasmons in CdTe-based QWs [25,26,27], we state that the resonances observed in Figure 6 on the etched sample were due to the excitation of a magnetoplasmon, which appeared not to be circularly polarized.

Taking into account that the state of polarization could be decomposed into circular and linear polarizations, one concludes that in the case of the sample with the etched grating, polarization of the magnetoplasmon was very close to linear. We noticed that the magnetotransmission spectra were not perfectly symmetrical under the inversion of the direction of *B*. This could arise either from a non-perfect experimental configuration mentioned above or from the presence of a small component of circular polarization in the MP. This possibility was indicated in [28] where the authors discussed the propagation of electromagnetic waves in the direction perpendicular to *B*. Their analysis showed that for a mode propagating with a frequency given by Equation (1) and $\omega_{c}∼\omega_{p}$, the propagating wave was not purely longitudinal, but contained also transverse components.

A seemingly weak point in our analysis was that the polarization properties of the etched gratings were studied at room temperature, while the polarization of MPs was tested at 2 K. The question is if the polarization properties of etched gratings can drastically change after cooling the sample. We think that this was not possible due to the following reasons. As one can conclude from comparison of etched and Au gratings, a grating can be a linear polarizer if it is metallic. This means that the concentration of electrons in the etched grating is too small to mimic a metal, at least at room temperature. To be more specific, the electron concentration in Au is of the order of $10^{23}$ cm^−3^, while the concentration of 2DEG is of the order of $10^{12}$ cm^−2^ in a quantum well of the width of the order of 10 nm. This corresponds to the bulk concentration of 2DEG of the order of $10^{18}$ cm^−3^, about five orders of magnitude less than in the case of Au. In the sample studied, with the Mg concentration in the barrier equal to 26%, the depth of the CdTe QWs was equal to about 255 meV, which was an order of magnitude higher than the energy of thermal excitations at 300 K. This meant that even at 300 K, free electrons were already confined in the QWs and the temperature had practically no impact on the electron distribution in the sample. Thus, etched gratings were not polarizing at low temperature, either.

A shift between the MP and CR resonances, which were observed at B=±9.105 T and B=±9.355 T, respectively, could be quantitatively described based on Equation (1) with a dispersion relation for ungated plasmons given by [29]:

(2) $$\omega_{p}=\sqrt{\frac{n_{s}e^{2}k}{2m^{*}\epsilon_{0}\epsilon \left(k\right)}},$$

where *k* is the wave vector of the (magneto)plasmon and the effective dielectric constant ϵ(k) is a *k*-dependent function, which in the case of ungated plasmons reads:

(3) $$\epsilon_{ug}\left(k\right)=\frac{1}{2}\left(\epsilon_{1}+\epsilon_{2}\frac{1+\epsilon_{2}\tanh\left(kd\right)}{\epsilon_{2}+\tanh\left(kd\right)}\right),$$

where *d* is the barrier thickness and $\epsilon_{1}$ and $\epsilon_{2}$ are dielectric constants of the quantum well (i.e., CdTe) and the barrier (i.e., CdMgTe), respectively.

**Figure 7:**
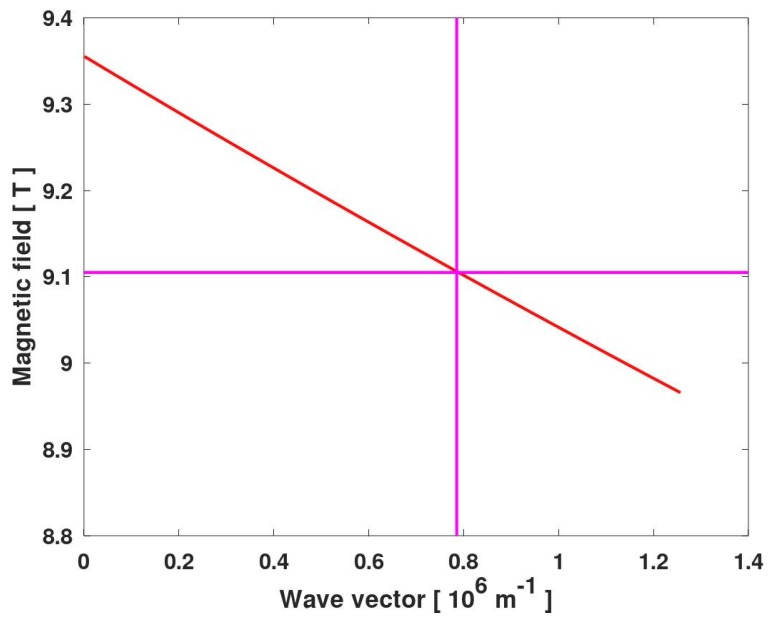
Dependence of the magnetic field at which an MP resonance occurs as a function of the MP wave vector (red line). The horizontal magenta line shows the magnetic field of 9.105 T at which the MP resonance was observed. The vertical magenta line shows the MP wave vector for Λ = 8 μm. Other parameters of the calculations are given in the main text.

In the case of samples with gratings of the period Λ, plasmon wave vectors can take values equal to an integer multiplicity of 2π/Λ, and we assumed that the resonance observed corresponded to the fundamental mode, i.e., k=2π/Λ. Taking together Equations (1)–(3) and the material parameters, one can calculate the magnetic field at which the MP resonance occurred. The results are presented by the red line in Figure 7. Let us note that this line points at k=0 to the observed magnetic field of the CR. It appeared that in this range of *k* and *B*, the dependence shown with the red line was linear, but in general, this is not the case.

In the analysis, it was assumed that the plasmons were ungated since there was neither a metallic gate, nor metallic grating that would screen the plasmon field. The complicated structure of the sample brought some difficulty in the estimation of the barrier thickness, *d*. The effective constant for ungated plasmons, given by Equation (3), was valid for the case of a 2DEG layer, which was surrounded by a semi-infinite dielectric substrate from one side and a dielectric layer of the thickness *d* from the other side. In the case of the sample studied, we dealt with two conducting layers, separated by a 44 nm thick CdMgTe layer, and the top barrier (of a thickness of 45 nm) was etched to form a grating. Apparently, the formula given by Equation (2) could not be, strictly speaking, applied to the sample studied, and the best solution would be to carry out numerical simulations of the system studied, which is, however, far beyond the scope of the present paper.

To deal with this problem, we assumed that the system could be described with Equation (3) with the effective parameters of dielectric constants, the concentration of 2DEG, and the barrier thickness. We took $n_{s}$ equal to $4.7\times 10^{11}$ cm^−2^, the concentration of 2DEG in each well determined by the magnetotransport experiments. We did not multiply this value by two because the two wells were separated with a thick spacer (44 nm of CdMgTe), and we assumed that plasmonic waves in these wells did not interact. We assumed a high frequency dielectric constant of CdTe $\epsilon_{1}=7.1$ [27], and we found that the results were weakly dependent on the value of the dielectric constant of CdMgTe, $\epsilon_{2}$. For this reason, we assumed that $\epsilon_{2}=\epsilon_{1}$. With this assumptions, we fitted *d* to get the red line in Figure 7 passing through the point of crossing of two magenta lines. It appeared that the best result, presented in Figure 7, was obtained with d=80±10 nm, which corresponded to the distance of the lower QW to the top surface of the sample. This result could indicate that etching of the structure led to a strong depopulation of the “upper” quantum well QW2 (see Figure 1), and plasmonic resonances in the “lower” quantum well QW1 were only visible. This conclusion should be, however, justified by a more detailed analysis of the influence of the etching profile on magnetoplasmon excitations.

## 5. Conclusions

We carried out polarization studies on CdTe-based quantum structures grown with molecular beam epitaxy and processed with electron-beam lithography. The samples contained two QWs, and their surface was covered either with a Au grating or a grating was wet-etched. Polarization studies at room temperature showed that Au gratings acted as a linear polarizer, but etched gratings were deprived of any polarization properties. Magnetotransmission experiments at 2 K with circularly polarized THz radiation showed that magnetoplasmon excitations were linearly polarized, although a small contribution from a circular polarization could not be excluded. Working with etched gratings, one can apply polarization experiments to distinguish between magnetoplasmon and cyclotron resonance referring to the polarization of a transmitted signal, and not only to a shift in magnetic field (between CR and MP). The present paper opens the possibility of more detailed studies of the polarization of magnetoplasmons in a 2DEG.

## Abbreviations

The following abbreviations are used in this manuscript:

2DEG	two-dimensional electron gas
AFM	atomic force microscope
CR	cyclotron resonance
MP	magnetoplasmon
PMMA	polymethyl methacrylate
QW	quantum well
SEM	scanning electron microscope
